# Individual visual working memory capacities and related brain oscillatory activities are modulated by color preferences

**DOI:** 10.3389/fnhum.2012.00318

**Published:** 2012-11-27

**Authors:** Masahiro Kawasaki, Yoko Yamaguchi

**Affiliations:** ^1^Rhythm-based Brain Information Processing Unit, RIKEN BSI-TOYOTA Collaboration CenterSaitama, Japan; ^2^Laboratory for Advanced Brain Signal Processing, RIKEN Brain Science InstituteSaitama, Japan; ^3^Neuroinformatics Japan Center, RIKEN Brain Science InstituteSaitama, Japan

**Keywords:** working memory, subjective preference, EEG, frontal, theta, beta

## Abstract

Subjective preferences affect many processes, including motivation, along with individual differences. Although incentive motivations are proposed to increase our limited visual working memory (VWM) capacity, much less is known about the effects of subjective preferences on VWM-related brain systems, such as the prefrontal and parietal cortices. Here, we investigate the differences in VWM capacities and brain activities during presentation of preferred and non-preferred colors. To this end, we used time-frequency (TF) analyses of electroencephalograph (EEG) data recorded during a delayed-response task. Behavioral results showed that the individual VWM capacities of preferred colors were significantly higher than those of non-preferred colors. The EEG results showed that the frontal theta and beta amplitudes for maintenance of preferred colors were higher than those of non-preferred colors. Interestingly, the frontal beta amplitudes were consistent with recent EEG recordings of the effects of reward on VWM systems, in that they were strongly and individually correlated with increasing VWM capacities from non-preferred to preferred colors. These results suggest that subjective preferences affect VWM systems in a similar manner to reward-incentive motivations.

## Introduction

As a well-known proverb says, “Everyone to his taste.” there are large individual differences in subjective preferences. Such preferences individually lead to either positive or negative emotions, which seem to influence not only our potential personalities, but also the current behaviors. However, little is known regarding whether subjective preferences directly affect cognitive processing. For example, Are we able to better memorize a preferred visual stimulus? To address this issue, it is useful to measure the visual working memory (VWM) capacity, which refers to our ability to memorize and maintain visual stimuli temporarily (Phillips, 1974; Pashler, 1988; Rensink, 2002). Previous psychological studies have suggested that VWM has a limited capacity and large individual differences, based on parametric load manipulation in a delayed matching-to-sample task, which required the short-term maintenance of several visual items (Luck and Vogel, 1997; Cowan, 2001; Vogel et al., 2001). The VWM capacity can be increased by training (Klingberg et al., 2002; Olesen et al., 2004; Jaeggi et al., 2008; McNab et al., 2009) or enhanced motivation by rewards such as money (Pochon et al., 2002; Gilbert and Fiez, 2004; Krawczyk et al., 2007; Kawasaki and Yamaguchi, in press). However, it not known whether VWM is affected by subjective preferences.

Many previous neuroimaging studies on humans have demonstrated that a vast network of brain regions—including the frontal, parietal, and visual cortices—forms the neural substrates for VWM (Courtney et al., 1997, 1998; Postle and D'Esposito, 1999; Pessoa et al., 2002). In particular, the frontal and parietal regions have been proposed to be associated with maintenance of mental representations. This is because activity in these areas is correlated with individual VWM capacity as demonstrated by electroencephalography (EEG) recordings (Gevins and Smith, 2000; Jensen and Tesche, 2002; Jensen et al., 2002; Vogel and Machizawa, 2004) and functional magnetic resonance imaging (fMRI) studies (Todd and Marois, 2005; Song and Jiang, 2006; Xu and Chun, 2006; Kawasaki et al., 2008; Cowan et al., 2011; Robitaille et al., 2011).

In contrast, the brain regions involved in preference decision-making are proposed to be part of the reward-related and emotion-related brain networks, as activities in these areas are enhanced during preference judgments, such as judging the attractiveness of faces or preferences for specific food and beverages. These networks are partially overlapped in the common brain regions including anterior frontal cortex, anterior cingulate, striatum, and amygdala (Aharon et al., 2001; O'Doherty et al., 2002; McClure et al., 2004). However, the emotion-related brain networks are more likely to include hedonic hotspots, such as the nucleus accumbens and ventromedial prefrontal cortex with opioid or cannabinoid neurotransmissions, whereas the reward-related brain networks are involved in the dopaminergic mid-brain and dopamine projected areas such as the medial orbitofrontal cortex (Berridge, 2003; Kringelbach and Berridge, 2009; Dai et al., 2010; Smillie et al., 2011). Indeed, enhanced ventromedial prefrontal activity is strongly correlated with individual subjective preferences, while the dopaminergic mid-brain and orbitofrontal cortex show correlation with the reward values (O'Doherty et al., 2002; Knutson et al., 2005). Moreover, recent fMRI studies have shown that the nucleus accumbens is involved in automatic and first impressions of preferences, whereas the orbitofrontal cortex is involved in decision-making (Kim et al., 2007).

Thus, although there is rich evidence for VWM- and preference-related brain activities individually, few studies have addressed the dynamic interactions between them. That is to say, there is little neurological evidence regarding how VWM-related networks are affected by differences in subjective preferences, although some studies have proposed that there are interactions between VWM- and reward-related brain activities (Pochon et al., 2002; Gilbert and Fiez, 2004; Krawczyk et al., 2007; McNab and Klingberg, 2008; Kawasaki and Yamaguchi, in press).

In this study, we investigated the effects of subjective preferences on VWM capacity and its neural mechanisms, using time-frequency (TF) analyses of EEG data. EEG oscillations are thought to reflect the synchronization of a large number of neurons underlying a particular function (Varela et al., 2001). In particular, low frequency-band activities such as the theta (4–8 Hz) and alpha (8–12 Hz) oscillations are thought to be related to several functions of VWM, such as the manipulation and maintenance of mental representations (Mizuhara et al., 2004; Sauseng et al., 2005; Klimesch et al., 2008; Kawasaki et al., 2010). Theta activity also seems to be related to emotional changes caused by subjective preferences, as theta activities in the frontal and occipital regions that are associated with pleasant stimuli are higher than those associated with unpleasant stimuli (Sammler et al., 2007; Lindsen et al., 2010; Kawasaki and Yamaguchi, 2012). Moreover, frontal beta activity is associated with motivation and the relative evaluation of reward values (Cohen et al., 2007; Marco-Pallares et al., 2008; Kawasaki and Yamaguchi, in press).

In this study, we focused on the individual subjective preferences for colors and compared the oscillatory behavior of the EEG under two conditions in a delayed response VWM task. In one condition, the subjects were required to memorize stimuli presented as colors that they preferred. In the other condition, we asked the subjects to memorize non-preferred colors. We hypothesized that subjective preferences would affect VWM processing, and both VWM capacity and associated EEG oscillation power would be reduced in the non-preferred condition.

## Materials and methods

Nineteen healthy right-handed volunteers (8 women, 11 men; mean age = 21.5 ± 0.5 years, range 18–27 years) took part in this experiment. They reported having normal or corrected-to-normal visual acuity, normal hearing acuity, and normal motor abilities using subjective questionnaires. All participants gave written informed consent, which was approved by the Ethical Committee of the RIKEN (in accordance with the Declaration of Helsinki), *prior* to participation in this study.

Before the EEG experiments, each participant completed a pretest to identify their personal color preferences (Kawasaki and Yamaguchi, 2012). In the pretest, participants were asked to choose between two colored squares presented in the right and left hemi-fields, relative to a central white fixation point on a 24-in. computer display (ProLite E2410HDS, Iiyama, JAPAN). Each trial consisted of 1 s of stimulus presentation, a 2 s response period for their judgment, and a 2 s inter-trial interval. Two colors were selected from the ten available colors [white (*r* = 255, *g* = 255, *b* = 255), red (255, 0, 0), green (0, 255, 0), blue (0, 0, 255), yellow (255, 255, 0), magenta (255, 0, 255), cyan (0, 255, 255), olive (128, 128, 0), purple (128, 0, 128), and aqua (0, 128, 128)]. Each participant completed 90 trials. All possible color combinations were presented twice, with a reshuffling of the right and left positions. We selected the most and least preferred colors for each individual using the number of times each color was selected.

**Figure 1:**
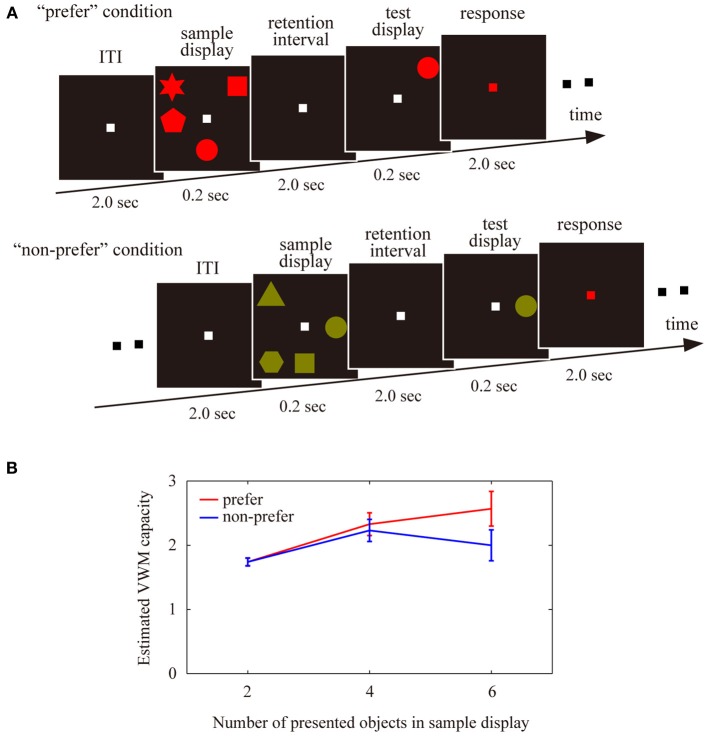
**(A)** Schematic display of the trial sequences used for the visual working memory tasks, in the “preferred” and “non-preferred” conditions. Participants were asked to memorize either 2, 4, or 6 colored shapes, to maintain these shaped in working memory during the retention intervals, and then to judge whether a single probe shape in a test display matched a sample disk in the same location. **(B)** Estimated visual working memory capacity under the “preferred” and “non-preferred” conditions with different numbers of presented objects (2, 4, or 6). Error bars represent the standard error of the mean.

In the VWM experiments, participants were required to memorize 2, 4, or 6 colored shapes (visual angle; approximately 1° × 1°, shape; circle, square, triangle, star, pentagon, parallelogram, cross, and trapezoid), which were simultaneously presented at random locations in an invisible 3 × 3 cell matrix for 0.2 s (Figure 1A, sample display). All colors were defined by the subject's favorite color (“preferred” condition) or least favorite color (“non-preferred” condition) in each trial. After a 2 s retention interval, the participants were required to judge whether a probe shape matched the shape at the same location in the sample display via a button press, while the fixation point was red for 2 s (test display). In half of the trials, the probe shape matched the sample shape. In the other half, the probe shape was replaced with another shape from the sample display. The inter-trial interval (ITI) was 2 s. Each participant completed three separate blocks consisting of 60 trials each, consisting of three shapes (2, 4, or 6) × 2 color preference conditions (“preferred” or “non-preferred”) × 2 change types (change or non-change of the probe shape from the sample shape) × 5 repetitions. Therefore, each condition (number of shapes × color preference) totaled 30 trials. All participants practiced in a behavioral training session before the EEG-measurement sessions. The training sessions were identical to the real sessions in their procedures and both had 60 trials.

EEG recordings were collected from 60 scalp electrodes (Ag/AgCl) embedded in an electro cap (Brain Cap; Brain Products, Germany) in accordance with the extended version of the international 10/20 system. Reference electrodes were placed on the right and left ear lobes. Electrooculography (EOG) measurements were recorded from electrodes above and below the left eye by monitoring eye blinks or vertical eye movements, and from electrodes placed 1 cm from the right and left eyes by monitoring horizontal eye movements. The EEG and EOG data were amplified using Neuroscan equipment (Compumedics NeuroScan Corp., Charlotte, NC, USA). The sampling rate was 500 Hz. The EEG data were filtered in the band-pass range from 0.1 to 50 Hz.

We segmented the EEG data into 3 s epochs (a 2 s retention interval and 0.5 s pre- and post-retention intervals; 1500 time points in total) for each trial. To reduce or eliminate artifacts, we conducted an infomax independent components analysis (ICA) on the EEG data from the correct trials. ICA components that significantly correlated with vertical or horizontal eye movements in the EOG data were rejected, and the ICA-corrected data were recalculated using a regression of the remaining components. To elucidate the cortical activity with decreasing errors from volume conduction, we applied current source density analysis using the spherical Laplace operator to the voltage distribution on the surface of the scalp (Perrin et al., 1989).

To identify the TF amplitudes during the retention interval, we applied wavelet transforms using Morlet's wavelets having a Gaussian shape in the time and the frequency domains around their center frequency (Tallon-Baudry et al., 1997). We used Morlet's wavelets for their high time and frequency resolutions, which allowed us to observe transitions in both the low and high frequency oscillations (Herrmann et al., 2005). The TF amplitude for each time point in each trial was the squared norm of the results of the convolution of the original EEG signals with a complex Morlet's wavelet function (*f*/σ_f_ = 7), ranging from 0.5 to 40 Hz in 0.5 Hz steps (i.e., 80 TF values at each time point for each single trial waveform). The TF amplitudes from the ITIs were averaged to generate the baseline amplitudes. The delay-period TF amplitude was calculated by subtracting the baseline data for each trial from each frequency band. The corrected TF amplitude was averaged across single trials for all conditions for each participant.

## Results

Accuracy rates (percentage correct) were higher when we presented fewer objects in both the “preferred” and “non-preferred” condition (2 objects, preferred: 94.0 ± 1.5%; 2 objects, non-preferred: 93.5 ± 1.6%; 4 objects, preferred: 79.1 ± 2.3%; 4 objects, non-preferred: 77.9 ± 2.2%; 6 objects, preferred: 71.2 ± 2.3%; 6 objects, non-preferred: 66.7 ± 2.1%). A Two-Way analysis of variance (ANOVA) revealed a main effect of the number of objects [*F*_(2, 108)_ = 72.69, *P* < 0.01], but no significant effect of preference [*F*_(1, 108)_ = 1.54, *P* = 0.22] and no significant interaction [*F*_(2, 108)_ = 0.54, *P* = 0.59].

The VWM capacity was estimated using Cowan's *K* formula: *K* = *N* × (hit rate + correct rejection rate—1), where *K* is the estimated number of objects stored in VWM, and *N* is the number of presented objects in the sample display (Cowan, 2001; Todd and Marois, 2004; Kawasaki et al., 2008). For each participant, each *K* value (*K*_2 objects_, *K*_4 objects_, and *K*_6 objects_) was calculated for each conditions for the 2 conditions (preferred or non-preferred) × the 3 different numbers of objects (2 or 4 or 6). To identify the limitation of the VWM capacity of each participant for each condition, we compared *K* values among 3 different numbers of objects and selected maximum *K* value (*K*_max_) for the preferred and non-preferred conditions. These methods were based on previous studies (Vogel and Machizawa, 2004; Todd and Marois, 2005).

The averaged VWM capacity for each condition is shown in Figure 1B. A Two-Way ANOVA revealed a significant interaction [*F*_(2, 108)_ = 6.87, *P* < 0.01], but no significant main effects of the number of objects [*F*_(2, 78)_ = 0.20, *P* = 0.81] or preference [*F*_(1, 78)_ = 1.00, *P* = 0.32]. There was a significant difference between the “preferred” and “non-preferred” conditions for the larger number of presented objects (2 objects, *Z* = 0.15, *P* = 0.88; 4 objects, *Z* = 0.78, *P* = 0.43; 6 objects, *Z* = 1.93, *P* < 0.05). The maximum VWM capacity, which was defined by the maximum values among all VWM capacities for the number of presented objects showed significant differences between the “preferred” and “non-preferred” conditions (*Z* = 2.44, *P* < 0.02; VWM capacity for preferred condition, 3.02 ± 0.16 objects; non-preferred condition, 2.62 ± 0.15 objects). These results suggest that subjective color-preference affected the available VWM capacity in our experiments.

**Figure 2:**
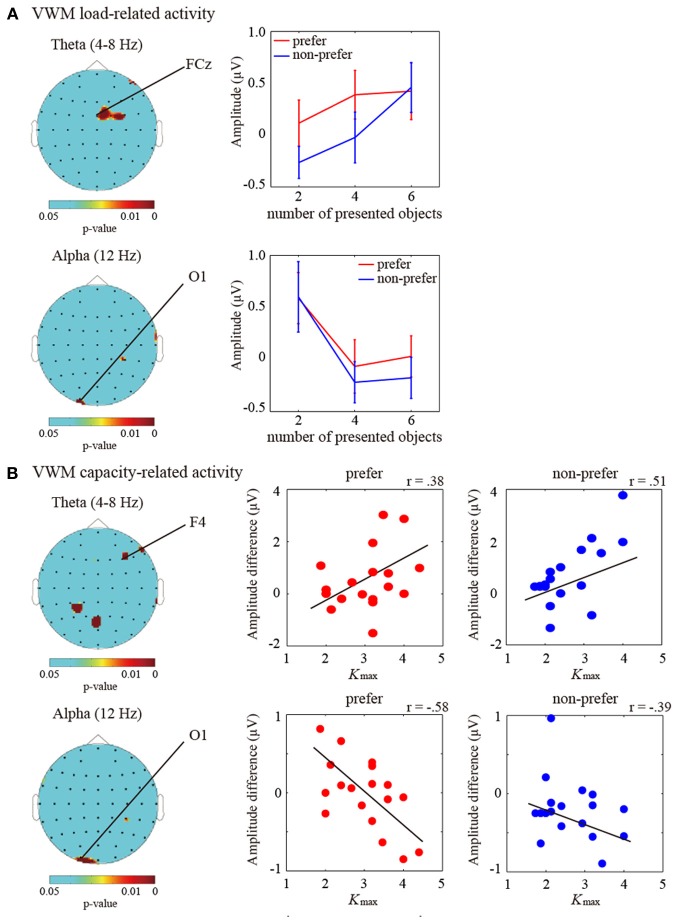
**(A)** (Left) Statistical topographic colored scalp maps of the theta (4–8 Hz; top panel) and alpha (12 Hz; bottom panel) delay-period activation showing p-values for the main effects of number of objects on ANOVA (*P* < 0.05). The drawing shows the top view of the scalp. (Right) Bar graph showing the subject-averaged amplitude of theta frequency bands at the frontal (FCz) electrode and alpha frequency bands at the occipital (O1) electrode under the “preferred” (red) and “non-preferred” (blue) conditions. Error bars represent standard errors of the mean. **(B)** (Left) Statistical topographic colored scalp maps showing *p*-values for the correlations between individual *K*_max_ values (VWM capacity estimates) and the theta (4–8 Hz; top panel) and alpha (12 Hz; bottom panel) delay-period activation differences between the *K*_max_ and two items (*P* < 0.05). (Right) Scatter plot showing the relationships between individual *K*_max_-values and individual theta and alpha amplitude differences between the *K*_max_ and two items at the frontal (F4) and occipital (O1) electrodes under the “preferred” and “non-preferred” conditions. Black lines represent the regression fit.

To identify the specific pattern of brain activity representing VWM maintenance, we calculated the TF amplitudes from the 60-channel EEG data during the 2 s retention intervals in comparison with the baseline periods. For the theta (the frequency: 4–8 Hz) and alpha (12 Hz) amplitudes, an ANOVA showed main effects of the number of objects in the frontal and parietal regions which electrodes were shown in Figure 2A (*P* < 0.05). The theta amplitudes in the frontal and right motor regions were enhanced with increasing number of objects, whereas the occipital alpha amplitudes showed opposite way.

Moreover, in order to investigate the VWM-capacity-related activities, we applied correlation analyses between the limitations of individual VWM capacities (i.e., maximum *K* values; *K*_max_) and individual differences in the amplitudes between *K*_max_ and *K*_2 objects_. Significant correlations were observed in the averaged theta (4–8 Hz) and alpha (12 Hz) activities (*P* < 0.05). The theta and alpha correlations were mainly found in the frontal/occipital and occipital regions, respectively. The topographic colored scalp maps for the significant statistical values are shown in Figure 2B. The frontal theta and occipital theta were positively correlated with individual VWM capacities [electrode measuring the peak statistic value, F4; preferred, *r*_(19)_ = 0.38, *P* < 0.05; non-preferred, *r*_(19)_ = 0.51, *P* < 0.01], whereas the occipital alpha amplitudes showed negative correlations [O1; preferred, *r*_(19)_ = −0.58, *P* < 0.01; non-preferred, *r*_(19)_ = −0.39, *P* < 0.05]. Although these statistical values were not enough for multiple comparisons, the frontal theta and occipital alpha oscillations are likely to be involved in the maintenance of VWM, which is similar to our previous findings.

**Figure 3:**
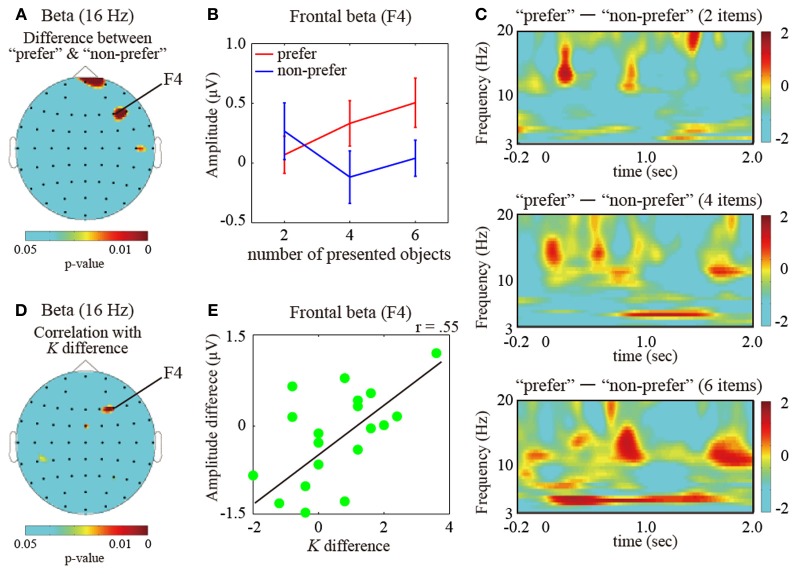
**(A)** Statistical topographic colored scalp maps showing *p*-values of the beta (16 Hz; frequency measuring the peak statistic value) activation differences between the “prefer” and “non-preferred” conditions. **(B)** Bar graph showing the subject-averaged amplitude of beta frequency bands at the frontal electrode (F4 electrode measuring the peak statistic value) under the “preferred” (red) and “non-preferred” (blue) conditions. Error bars represent standard errors of the mean. **(C)** Different time-frequency amplitudes during the retention intervals for “preferred” and “non-preferred” conditions for the maintenance of 2 (top), 4 (middle), and 6 (bottom) items measured at the F4 electrode. These values were normalized with respect to the inter-trial interval baseline and averaged across successful trials for all participants. The dotted vertical lines indicate the onset of the retention interval. **(D)** Topographic colored scalp maps showing *p*-values for the beta delay-period amplitudes, which were significantly correlated with increasing VWM capacity between the “preferred” and “non-preferred” conditions. **(E)** Scatter plot showing different VWM capacities and F4 electrode beta amplitudes between the “preferred” and “non-preferred” conditions.

Next, to investigate the pattern of brain activity representing the effects of color preference on VWM capacity, we compared delay-period oscillatory amplitudes between the “preferred” and “non-preferred” conditions under the high VWM load (6 objects), where the VWM capacity was found to be significantly different between the preference conditions. For the theta amplitudes (4–8 Hz), significant differences in amplitude were found in the frontal, parietal, and occipital brain regions (multiple comparison test with Bonferroni correction for the number of electrodes (i.e., comparison was 61); frontal (AF8 electrodes), *P* < 0.05; parietal (Pz), *P* < 0.05; occipital (O1), *P* < 0.05). In addition, low beta activities (12–20 Hz) under the “preferred” condition were significantly higher than under the “non-preferred” condition, and the enhancements were distributed across both the right lateral frontal areas (F4, *P* < 0.05) and the anterior frontal areas (Fpz, *P* < 0.05) (Figures 3A,B). However, significant differences were not found in the alpha ranges.

The time-course transitions of the different amplitudes between the “preferred” and “non-preferred” conditions on the right frontal electrode (F4) during the maintenance of 2, 4, and 6 items are shown in Figure 3C (top, middle, and bottom, respectively). The frontal low beta amplitudes, including the alpha amplitudes, were discretely higher during the retention intervals under the “preferred” condition. These enhancements were large and long-lasting, increasing with an increasing number of items in the sample display. On the other hand, the frontal theta enhancements were more sustained during the retention interval for six objects under the “preferred” condition in comparison to the “non-preferred” condition.

Finally, to identify the brain oscillations reflecting increased VWM capacity under the “preferred” condition, we examined the activity patterns under the “preferred” condition as compared to those under the “non-preferred” condition. Frontal beta (16 Hz; frequency measuring the peak statistic value) delay-period amplitudes were significantly correlated with the increased VWM capacity in preferred colors vs. non-preferred colors conditions [see Figures 3D,E; electrode measuring the peak statistic value, F4: *r*_(19)_ = 0.55, *P* < 0.01]. The frontal areas overlapped the VWM capacity-correlated regions. For the other VWM-capacity-correlated regions, the frontal theta and occipital alpha amplitudes did not significantly correlate with preference-induced increases in VWM capacity [F4 theta: *r*_(19)_ = 0.13, *P* = 0.41; O1 alpha: *r*_(14)_ = −0.09, *P* = 0.62].

## Discussion

The current study clearly shows that, subjective preferences for visual stimuli affect VWM capacity in individuals. VWM capacity was significantly higher with a subject's favorite color compared to their less preferred colors, particularly under conditions involving high VWM loads. This result is in agreement with previous findings showing that VWM capacity is enhanced by anticipation of high monetary rewards for correct answers, in comparison with low or no monetary rewards (Pochon et al., 2002; Gilbert and Fiez, 2004; Krawczyk et al., 2007; Kawasaki and Yamaguchi, in press). Therefore, subjectively preferred stimuli *per se* may affect VWM systems, much like the anticipation of reward.

In relation to the behavioral results, our EEG results demonstrate that VWM-related brain activities are modulated by subjective preferences. First, we identified delay-period theta and alpha oscillatory activities in the frontal and occipital regions, which are related to number of objects to be remembered (VWM load) and strongly correlated with individual differences in VWM capacities, as patterns of brain activity directly related to VWM. These VWM-load and VWM-capacity-related brain mechanisms were not overlapped. These results might support the previous fMRI finding about functional dissociations between VWM-load and VWM-capacity-related regions in the fronto-parietal networks (Linden et al., 2003), although EEG has spatial limitations in comparison with their fMRI. Moreover, the frontal theta and occipital alpha activities showed opposing relationships to VWM capacity, as frontal theta activity was positively correlated and occipital alpha activity negatively correlated with VWM capacity. These results are somewhat consistent with previous findings using fMRI (Linden et al., 2003; Todd and Marois, 2004, 2005; Song and Jiang, 2006; Xu and Chun, 2006; Kawasaki et al., 2008; Cowan et al., 2011; Robitaille et al., 2011) and EEG recording (Gevins et al., 1979; Jensen and Tesche, 2002; Jensen et al., 2002; Klimesch et al., 2008; Sauseng et al., 2009), even though many fMRI studies reported positive correlations between parietal blood oxygenation level dependent (BOLD) signals and VWM capacities. However, the negative correlations between occipital alpha activity and VWM capacity in the current study may well be because of negative relationships between the BOLD signals and alpha activities (Goldman et al., 2002; Laufs et al., 2003; Moosmann et al., 2003; Meltzer et al., 2007; Michels et al., 2008).

Thus, the frontal and occipital regions are candidate neural substrates for the maintenance of VWM, in agreement with a number of previous electrophysiological studies in non-human primates (e.g., Friedman and Goldman-Rakic, 1994) and many fMRI studies in humans (e.g., Curtis and D'Esposito, 2003). Previous EEG studies have also reported that theta and alpha activities in extended brain regions increase during several VWM tasks, including delayed matching-to-sample, *n*-back, mental manipulation, spatial WM, and mental calculation tasks (Ishihara and Yoshii, 1972; Tesche and Karhu, 2000; Kahana et al., 2001; Raghavachari et al., 2001; Busch and Herrmann, 2003; Cooper et al., 2003; Mizuhara et al., 2004; Klimesch et al., 2005; Kawasaki and Watanabe, 2007; Klimesch et al., 2008; Sauseng et al., 2009; Kawasaki et al., 2010).

VWM-related frontal activities are affected by subjective preferences, as demonstrated by the oscillatory amplitudes correlated with VWM capacity being enhanced under the “preferred” condition as compared to the “non-preferred” condition. The effects of preference on theta amplitudes were strongly and sustainably observed during the retention intervals in this study. These results suggest that the frontal oscillations may reflect the motivational effects of subjective preferences via signals from the reward- and/or emotion-related brain regions during the maintenance of VWM, although these brain regions are proposed to be separated (Berridge, 2003; Kringelbach and Berridge, 2009; Dai et al., 2010; Smillie et al., 2011).

On the other hand, frontal beta activities play an important role in the facilitation of VWM systems by subjective preferences, much as they do with reward incentive motivations. Indeed, beta amplitudes were significantly correlated with increasing VWM capacity from the “non-preferred” to “preferred” conditions, which were similar to previous findings showing improvements in VWM capacity with increasing monetary reward (Kawasaki and Yamaguchi, in press). The beta activities are unlikely to be involved in the enhancements of subjective preferences themselves, because the beta activities did not show any significant differences between the preferred and non-preferred colors during the preference judgment tasks in our recent study (Kawasaki and Yamaguchi, 2012). Moreover, frontal beta activities transiently increased during maintenance of a preferred color, in comparison with non-preferred colors. The modulated activities are distributed not only among lateral parts (F4 and F6 electrodes) but also among anterior parts (Fpz and Fp2 electrodes) of the frontal regions. The anterior and lateral frontal regions are thought to be involved in judgments of preference (Aharon et al., 2001; O'Doherty et al., 2002; McClure et al., 2004).

The preference-related beta activities might be involved in opioid or cannabinoid neurotransmissions which play an important role in processing of emotion (Berridge, 2003; Kringelbach and Berridge, 2009). In contrast, the beta activities were also reported in the similar brain regions during reward predictions (Elliott et al., 2000; Knutson et al., 2001; O'Doherty et al., 2002; Gottfried et al., 2003; McClure et al., 2003; Knutson et al., 2004; Kable and Glimcher, 2007) and the presentation of monetary reward magnitudes and their probabilities, relative to loss feedback for gambling (Marco-Pallares et al., 2008) and reinforcement learning tasks (Cohen et al., 2007). These enhanced frontal beta activities may be related to the mid-brain dopaminergic responses and striatal activities, because the durations of these beta amplitudes are similar to the time-course of frontal dopamine-related activity (Fiorillo et al., 2003; McClure et al., 2003; Schultz, 2007). Moreover, the dopamine-related activity is proposed to be related to the different personality traits which would be tightly linked to subjective preferences in the present study (Depue and Collins, 1999; Zald et al., 2008; Previc, 2009). Considering these data together, frontal beta activities seem to be related to individual different signals of motivation derived from not only reward-related brain regions bus also emotion-related brain regions. However, it is worth noting that EEG studies have inherent limitations in identifying the precise source of beta activity. Therefore, it is important to identify the detailed neural networks involved in motivation in future studies, possibly by making use of simultaneous fMRI and EEG.

In contrast to the frontal regions, occipital regions are involved in VWM maintenance, irrespective of subjective preferences, since the VWM-capacity-related alpha activities showed no differences between the “preferred” and “non-preferred” conditions. However, it is possible that the occipital alpha decrements were affected by the amount of visual processing, since we did not include a control condition requiring participants to merely look at but not to memorize the visual stimuli, as was included in previous studies (e.g., Todd and Marois, 2004). Indeed, the occipital regions contain various visual cortices. However, the occipital theta amplitudes for the “preferred” condition were higher than those for the “non-preferred” condition, even though the activity patterns did not directly represent individual VWM capacities. This may be explained by previous findings that attention-related occipital theta activities were enhanced by subjective preferences (Kawasaki and Yamaguchi, 2012), because VWM tasks require visual attention (Awh and Jonides, 2001).

In this study, it should be noted that there remains the possibility that not only subjective preferences but also other factors such as differences between the discrimination and familiarity of colors may affect VWM capacity and EEG differences (e.g., brightness, RBC dimensions and so on), because the chosen colors differed between the participants. (Preferred color: red, 3; green, 1; blue, 4; yellow, 1; magenta, 1; cyan, 3; olive, 0; purple, 1; aqua, 3; white, 2) (Non-preferred color: red, 3; green, 0; blue, 0; yellow, 1; magenta, 1; cyan, 2; olive, 7; purple, 1; aqua, 1; white, 3). Therefore, future study should rigorously clarify such effects of the color components on VWM capacity and EEG activities.

This study focused on the influence of the color preference on VWM capacity, however, visual stimulus included several features such as shapes. There are possibilities that preferred shapes are better memorized than non-preferred shapes. Unfortunately, it is difficult to judge such preferences of shapes in this study. So, it is necessary to confirm the effects of preference on the VWM capacity by using other visual features in future study.

### Conflict of interest statement

The authors declare that the research was conducted in the absence of any commercial or financial relationships that could be construed as a potential conflict of interest.

## References

[B1] Aharon I. Etcoff N. Ariely D. Chabris C. F. O'Connor E. Breiter H. C. (2001). Beautiful faces have variable reward value: fMRI and behavioral evidence. Neuron 32, 537–551 10.1016/S0896-6273(01)00491-3 11709163

[B2] Awh E. Jonides J. (2001). Overlapping mechanisms of attention and spatial working memory. Trends Cogn. Sci. 5, 119–126 10.1016/S1364-6613(00)01593-X 11239812

[B3] Berridge K. C. (2003). Pleasures of the brain. Brain Cogn. 52, 106–128 10.1016/S0278-2626(03)00014-9 12812810

[B4] Busch N. A. Herrmann C. S. (2003). Object-load and feature-load modulate EEG in a short-term memory task. Neuroreport 14, 1721–1724 10.1097/01.wnr.0000087727.58565.1b 14512845

[B5] Cohen M. X. Elger C. E. Ranganath C. (2007). Reward expectation modulates feedback-related negativity and EEG spectra. Neuroimage 35, 968–978 10.1016/j.neuroimage.2006.11.056 17257860 PMC1868547

[B6] Cooper N. R. Croft R. J. Dominey S. J. J. Burgess A. P. Gruzelier J. H. (2003). Paradox lost? Exploring the role of alpha oscillations during externally vs. internally directed attention and the implications for idling and inhibition hypotheses. Int. J. Psychophysiol. 47, 65–74 10.1016/S0167-8760(02)00107-1 12543447

[B7] Courtney S. M. Petit L. Maisog J. M. Ungerleider L. G. Haxby J. V. (1998). An area specialized for spatial working memory in human frontal cortex. Science 279, 1347–1351 10.1162/089892900564073 9478894

[B8] Courtney S. M. Ungerleider L. G. Keil K. Haxby J. V. (1997). Transient and sustained activity in a distributed neural system for human working memory. Nature 386, 608–611 10.1038/386608a0 9121584

[B9] Cowan N. (2001). The magical number 4 in short-term memory: a consideration of mental storage capacity. Behav. Brain Sci. 24, 87–114 11515286 10.1017/s0140525x01003922

[B10] Cowan N. Li D. Moffitt A. Becker T. M. Martin E. A. Saults J. S. (2011). A neural region of abstract working memory., J. Cogn. Neurosci. 23, 2852–2863 10.1162/jocn.2011.21625 21261453 PMC3138911

[B11] Curtis C. E. D'Esposito M. (2003). Persistent activity in the prefrontal cortex during working memory. Trends Cogn. Sci. 7, 415–423 10.1016/S1364-6613(03)00197-9 12963473

[B12] Dai X. Brendl C. M. Ariely D. (2010). Wanting, liking, and preference construction. Emotion 10, 324–334 10.1037/a0017987 20515222

[B13] Depue R. A. Collins P. F. (1999). Neurobiology of the structure of personality: dopamine, facilitation of incentive motivation, and extraversion. Behav. Brain Sci. 22, 491–517 11301519 10.1017/s0140525x99002046

[B14] Elliott R. Friston K. J. Dolan R. J. (2000). Dissociable neural responses in human reward systems. J. Neurosci. 20, 6159–6165 10934265 10.1523/JNEUROSCI.20-16-06159.2000 PMC6772605

[B15] Fiorillo C. D. Tobler P. N. Schultz W. (2003). Discrete coding of reward probability and uncertainty by dopamine neurons. Science 299, 1898–1902 10.1126/science.1077349 12649484

[B16] Friedman H. R. Goldman-Rakic P. S. (1994). Coactivation of prefrontal cortex and inferior parietal cortex in working memory tasks revealed by 2DG functional mapping in the rhesus monkey. J. Neurosci. 14, 2775–2788 8182439 10.1523/JNEUROSCI.14-05-02775.1994 PMC6577453

[B17] Gevins A. S. Zeitlin G. M. Doyle J. C. Yingling C. D. Schaffer R. E. Callaway E. (1979). Electroencephalogram correlates of higher cortical functions. Science 203, 665–668 10.1126/science.760212 760212

[B18] Gevins A. Smith M. (2000). Neurophysiological measures of working memory and individual differences in cognitive ability and cognitive style. Cereb. Cortex 10, 829–839 10982744 10.1093/cercor/10.9.829

[B19] Gilbert A. M. Fiez J. A. (2004). Integrating rewards and cognition in the frontal cortex. Cogn. Affect. Behav. Neurosci. 4, 540–552 15849896 10.3758/cabn.4.4.540

[B20] Goldman R. I. Stern J. M. Engel J. Jr. Cohen M. S. (2002). Simultaneous EEG and fMRI of the alpha rhythm. Neuroreport 13, 2487–2492 10.1097/01.wnr.0000047685.08940.d0 12499854 PMC3351136

[B21] Gottfried J. A. O'Doherty J. Dolan R. J. (2003). Encoding predictive reward value in human amygdala and orbitofrontal cortex. Science 301, 1104–1107 10.1126/science.1087919 12934011

[B22] Herrmann C. S. Grigutsch M. Busch N. A. (2005). EEG oscillations and wavelet analysis, in Event-Related Potentials: A Methods Handbook, ed Handy T. C. (Cambridge, MA: MIT Press), 229–259

[B23] Ishihara T. Yoshii N. (1972). Multivariate analytic study of EEG and mental activity in juvenile delinquents. Electroencephalogr. Clin. Neurophysiol. 33, 71–80 4113276 10.1016/0013-4694(72)90026-0

[B24] Jaeggi S. M. Buschkuehl M. Jonides J. Perrig W. J. (2008). Improving fluid intelligence with training on working memory. PNAS 105, 6829–6833 10.1073/pnas.0801268105 18443283 PMC2383929

[B25] Jensen O. Tesche C. (2002). Frontal theta activity in humans increases with memory load in a working memory task. Eur. J. Neurosci. 15, 1395–1400 10.1046/j.1460-9568.2002.01975.x 11994134

[B26] Jensen O. Gelfand J. Kounious K. Lisman J. E. (2002). Oscillations in the alpha band (9–12 Hz) increase with memory load during retention in a short-term memory task. Cereb. Cortex 12, 877–882 12122036 10.1093/cercor/12.8.877

[B27] Kable J. W. Glimcher P. W. (2007). The neural correlates of subjective value during intertemporal choice. Nat. Neurosci. 10, 1625–1633 10.1038/nn2007 17982449 PMC2845395

[B28] Kahana M. J. Seelig D. Madsen J. R. (2001). Theta returns. Curr. Opin. Neurobiol. 11, 739–744 10.1016/j.ijpsycho.2005.06.003 11741027

[B29] Kawasaki M. Watanabe M. (2007). Oscillatory gamma and theta activity during repeated mental manipulations of a visual image. Neurosci. Lett. 422, 141–145 10.1016/j.neulet.2007.04.079 17602835

[B30] Kawasaki M. Yamaguchi Y. (2012). Effects of subjective preferences of colors on attention-related occipital theta oscillations. Neuroimage 59, 808–814 10.1016/j.neuroimage.2011.07.042 21820064

[B31] Kawasaki M. Yamaguchi Y. (in press). Frontal theta and beta synchronizations for monetary reward increase visual working memory capacity. Soc. Cogn. Affect. Neurosci. 10.1093/scan/nss027 22349800 PMC3682435

[B32] Kawasaki M. Kitajo K. Yamaguchi Y. (2010). Dynamic links between theta executive functions and alpha storage buffers in auditory and visual working memory. Eur. J. Neurosci. 31, 1683–1689 10.1111/j.1460-9568.2010.07217.x 20525081 PMC2878597

[B33] Kawasaki M. Watanabe M. Okuda J. Sakagami M. Aihara K. (2008). Human posterior parietal cortex maintains color, shape and motion in visual short-term memory. Brain Res. 1213, 91–97 10.1016/j.brainres.2008.03.037 18455152

[B34] Kim H. Adolphs R. O'Doherty J. P. Shimojo S. (2007). Temporal isolation of neural processes underlying face preference decisions. PNAS 104, 18253–18258 10.1073/pnas.0703101104 17989234 PMC2084329

[B35] Klimesch W. Freunberger R. Sauseng P. Gruber W. (2008). A short review of slow phase synchronization and memory: evidence for control processes in different memory systems? Brain Res. 1235, 31–44 10.1016/j.brainres.2008.06.049 18625208

[B36] Klimesch W. Schack B. Sauseng P. (2005). The functional significance of theta and upper alpha oscillations. Exp. Psychol. 52, 99–108 15850157 10.1027/1618-3169.52.2.99

[B37] Klingberg T. Forssberg H. Westerberg H. (2002). Training of working memory in children with ADHD. J. Clin. Exp. Neuropsychol. 24, 781–791 10.1076/jcen.24.6.781.8395 12424652

[B38] Knutson B. Bjork J. M. Fong G. W. Hommer D. W. Mattay V. S. Weinberger D. R. (2004). Amphetamine modulates human incentive processing. Neuron 43, 261–269 10.1016/j.neuron.2004.06.030 15260961

[B39] Knutson B. Fong G. W. Adams C. M. Varner J. L. Hommer D. (2001). Dissociation of reward anticipation and outcome with event-related fMRI. Neuroreport 12, 3683–3687 11726774 10.1097/00001756-200112040-00016

[B40] Knutson B. Taylor J. Kaufman M. Peterson R. Glover G. (2005). Distributed neural representation of expected value. J. Neurosci. 25, 4806–4812 10.1523/JNEUROSCI.0642-05.2005 15888656 PMC6724773

[B41] Krawczyk D. C. Gazzaley A. D'Esposito M. (2007). Reward modulation of prefrontal and visual association cortex during an incentive working memory task. Brain Res. 1141, 168–177 10.1016/j.brainres.2007.01.052 17320835

[B42] Kringelbach M. L. Berridge K. C. (2009). Towards a functional neuroanatomy of pleasure and happiness. Trends Cogn. Sci. 13, 479–487 10.1016/j.tics.2009.08.006 19782634 PMC2767390

[B43] Laufs H. Kleinschmidt A. Beyerle A. Eger E. Salek-Haddadi A. Preibisch C. (2003). EEG-correlated fMRI of human alpha activity. Neuroimage 19, 1463–1476 10.1016/S1053-8119(03)00286-6 12948703

[B44] Linden D. E. J. Bittner R. A. Muckli L. Waltz J. A. Kriegeskorte N. Goebel R. (2003). Cortical capacity constraints of visual working memory: dissociation of fMRI load effects in a front-parietal network. Neuroimage 20, 1518–1530 10.1016/j.neuroimage.2003.07.021 14642464

[B45] Lindsen J. P. Jones R. Shimojo S. Bhattacharya J. (2010). Neural components underlying subjective preferential decision making. Neuroimage 50, 1626–1632 10.1016/j.neuroimage.2010.01.079 20116436

[B46] Luck S. J. Vogel E. K. (1997). The capacity of visual working memory for features and conjunctions. Nature 390, 279–281 10.1038/36846 9384378

[B47] Marco-Pallares J. Cucurell D. Cunillera T. García R. Andrés-pueyo A. Münte T. F. (2008). Human oscillatory activity associated to reward processing in a gambling task. Neuropsychologia 46, 241–248 10.1016/j.neuropsychologia.2007.07.016 17804025

[B48] McClure S. M. Berns G. S. Montague P. R. (2003). Temporal prediction errors in a passive learning task activate human striatum. Neuron 38, 339–346 10.1016/S0896-6273(03)00154-5 12718866

[B49] McClure S. M. Li J. Tomlin D. Cypert K. S. Montague L. M. Montague P. R. (2004). Neural correlates of behavioral preference for culturally familiar drinks. Neuron 44, 379–387 10.1016/j.neuron.2004.09.019 15473974

[B50] McNab F. Klingberg T. (2008). Prefrontal cortex and basal ganglia control access to working memory. Nat. Neurosci. 11, 103–107 10.1038/nn2024 18066057

[B51] McNab F. Varrone A. Farde L. Jucaite A. Bystritsky P. Forssberg H. (2009). Changes in cortical dopamine D1 receptor binding associated with cognitive training. Science 323, 800–802 10.1126/science.1166102 19197069

[B52] Meltzer J. A. Negishi M. Mayes L. C. Constable R. T. (2007). Individual differences in EEG theta and alpha dynamics during working memory correlate with fMRI responses across subjects. Clin. Neurophysiol. 118, 2419–2436 10.1016/j.clinph.2007.07.023 17900976 PMC2080790

[B53] Michels L. Moazami-Goudarzi M. Jeanmonod D. Sarnthein J. (2008). EEG alpha distinguishes between cuneal and precuneal activation in working memory. Neuroimage 40, 1296–1310 10.1016/j.neuroimage.2007.12.048 18272404

[B54] Mizuhara H. Wang L. Q. Kobayashi K. Yamaguchi Y. (2004). A long-range cortical network emerging with theta oscillation in a mental task. Neuroreport 15, 1233–1238 15167540 10.1097/01.wnr.0000126755.09715.b3

[B55] Moosmann M. Ritter P. Krastel I. Brink A. Thees S. Blankenburg F. (2003). Correlates of alpha rhythm in functional magnetic resonance imaging and near infrared spectroscopy. Neuroimage 20, 145–158 10.1016/S1053-8119(03)00344-6 14527577

[B56] O'Doherty J. P. Deichmann R. Critchley H. D. Dolan R. J. (2002). Neural responses during anticipation of a primary taste reward. Neuron 33, 815–826 10.1016/S0896-6273(02)00603-7 11879657

[B57] Olesen P. J. Westerberg H. Klingberg T. (2004). Increase prefrontal and parietal activity after training of working memory. Nat. Neurosci. 7, 75–79 10.1038/nn1165 14699419

[B58] Pashler H. (1988). Familiarity and visual change detection. Percept. Psychophys. 44, 369–378 3226885 10.3758/bf03210419

[B59] Perrin F. Pernier J. Bertrand O. Echallier J. F. (1989). Spherical splines for scalp potential and current density mapping. Electroencephalogr. Clin. Neurophysiol. 72, 184–187 2464490 10.1016/0013-4694(89)90180-6

[B60] Pessoa K. Gutierrez E. Bandettini P. A. Ungerleider L. G. (2002). Neural correlates of visual working memory: fMRI amplitude predicts task performance. Neuron 35, 975–987 10.1016/S0896-6273(02)00817-6 12372290

[B61] Phillips W. A. (1974). On the distinction between sensory storage and short-term visual memory. Percept. Psychophys. 16, 283–290

[B62] Pochon J. B. Levy R. Fossati P. Lehericy S. Poline J. B. Pillon B. (2002). The neural system that bridges reward and cognition in humans: an fMRI study. PNAS 99, 5669–5674 10.1073/pnas.082111099 11960021 PMC122829

[B63] Postle B. R. D'Esposito M. (1999). “What”—then—“Where” in visual working memory: an event-related fMRI study. J. Cogn. Neurosci. 11, 585–597 10601740 10.1162/089892999563652

[B64] Previc F. (2009). The Dopaminergic Mind in Human Evolution and History. Cambridge, UK: Cambridge University Press

[B65] Raghavachari S. Kahana M. Rizzuto D. Caplan J. Kirschen M. Burgeois B. (2001). Gating of human theta oscillations by a working memory task. J. Neurosci. 21, 3175–3183 11312302 10.1523/JNEUROSCI.21-09-03175.2001 PMC6762557

[B66] Rensink R. A. (2002). Change detection. Annu. Rev. Psychol. 53, 245–277 10.1146/annurev.psych.53.100901.135125 11752486

[B67] Robitaille N. Marois R. Todd J. Grimault S. Cheyne D. Jolicoeur P. (2011). Distinguishing between lateralized and nonlateralized brain activity associated with visual short-term memory: fMRI, MEG, and EEG evidence from the same observers. Neuroimage 53, 1334–1345 10.1016/j.neuroimage.2010.07.027 20643214

[B68] Sammler D. Grigutsch M. Fritz T. Koelsch S. (2007). Music and emotion: electrophysiological correlates of the processing of pleasant and unpleasant music. Psychophysiology 44, 293–304 10.1111/j.1469-8986.2007.00497.x 17343712

[B69] Sauseng P. Klimesch W. Heise K. Gruber W. R. Holz E. M. Glennon M. (2009). Brain oscillatory substrates of visual short-term memory capacity. Curr. Biol. 19, 1846–1852 10.1016/j.cub.2009.08.062 19913428

[B70] Sauseng P. Klimesch W. Stadler W. Schabus M. Doppelmayr M. Hanslmayr S. (2005). A shift of visual spatial attention is selectively associated with human EEG alpha activity. Eur. J. Neurosci. 22, 2917–2926 10.1111/j.1460-9568.2005.04482.x 16324126

[B71] Schultz W. (2007). Behavioral dopamine signals. Trends Neurosci. 30, 203–210 10.1016/j.tins.2007.03.007 17400301

[B72] Smillie L. D. Loxton N. J. Avery R. E. (2011). Reinforcement sensitivity theory, research, applications and future, in Handbook of Individual Differences, eds Chamorro-Premuzic T. von Stum S. Furnham A. (Oxford: Wiley-Blackwell), 101–131

[B73] Song J.-H. Jiang Y. (2006). Visual working memory for simple and complex features: an fMRI study. Neuroimage 30, 963–972 10.1016/j.neuroimage.2005.10.006 16300970

[B74] Tallon-Baudry C. Bertrand O. Delpuech C. Pernier J. (1997). Oscillatory gamma band activity (30–70 Hz) induced by a visual search task in human. J. Neurosci. 17, 722–734 8987794 10.1523/JNEUROSCI.17-02-00722.1997 PMC6573221

[B75] Tesche C. Karhu J. (2000). Theta oscillations index human hippocampal activation during a working memory task. PNAS 97, 919–924 10.1073/pnas.97.2.919 10639180 PMC15431

[B76] Todd J. J. Marois R. (2004). Capacity limit of visual short-term memory in human posterior parietal cortex. Nature 428, 751–754 10.1016/j.neuropsychologia.2009.12.005 15085133

[B77] Todd J. J. Marois R. (2005). Posterior parietal cortex activity predicts individual differences in visual short-term memory capacity. Cogn. Affect. Behav. Neurosci. 5, 144–155 16180621 10.3758/cabn.5.2.144

[B78] Varela F. Lachaux J. P. Rodriguez E. Martinerie J. (2001). The brainweb: phase synchronization and large-scale integration. Nat. Rev. Neurosci. 2, 229–239 10.1038/35067550 11283746

[B79] Vogel E. K. Machizawa M. G. (2004). Neural activity predicts individual differences in visual working memory capacity. Nature 428, 748–751 10.1162/jocn_a_00107 15085132

[B80] Vogel E. K. Woodman G. F. Luck S. J. (2001). Storage of features, conjunctions, and objects in visual working memory. J. Exp. Psychol. Hum. Percept. Perform. 27, 92–114 10.1037/0096-1523.27.1.92 11248943

[B81] Xu Y. Chun M. M. (2006). Dissociable neural mechanisms supporting visual short-term memory for objects. Nature 440, 91–95 10.1038/nature04262 16382240

[B82] Zald D. H. Cowan R. L. Riccardi P. Baldwin R. M. Ansari M. S. Li R. (2008). Midbrain dopamine receptor availability is inversely associated with novelty-seeking traits in humans. J. Neurosci. 28, 14372–14378 10.1523/JNEUROSCI.2423-08.2008 19118170 PMC2748420

